# Reversal of cancer gene expression identifies repurposed drugs for diffuse intrinsic pontine glioma

**DOI:** 10.1186/s40478-022-01463-z

**Published:** 2022-10-23

**Authors:** Guisheng Zhao, Patrick Newbury, Yukitomo Ishi, Eugene Chekalin, Billy Zeng, Benjamin S. Glicksberg, Anita Wen, Shreya Paithankar, Takahiro Sasaki, Amreena Suri, Javad Nazarian, Michael E. Pacold, Daniel J. Brat, Theodore Nicolaides, Bin Chen, Rintaro Hashizume

**Affiliations:** 1 Department of Pediatrics, New York University Langone Health, 160 East 32nd St., New York, NY 10016 USA; 2 Department of Pediatrics and Human Development, Michigan State University, Secchia Center, Room 732, 15 Michigan St. NE, Grand Rapids, MI 49503 USA; 3 Department of Pediatrics, Northwestern University Feinberg School of Medicine, 303 East Superior St., Simpson Querrey 4-514, Chicago, IL 60611 USA; 4 Division of Hematology, Oncology, Neuro-Oncology & Stem Cell Transplantation, Ann & Robert H. Lurie Children’s Hospital of Chicago, 225 East Chicago Avenue, Box 205, Chicago, IL 60611 USA; 5 Department of Genetics and Genomic Sciences, Icahn School of Medicine at Mount Sinai, 1 Gustave L. Levy Place, New York, NY 10029 USA; 6 Icahn School of Medicine at Mount Sinai, Hasso Plattner Institute for Digital Health at Mount Sinai, 1 Gustave L. Levy Place, New York, NY 10029 USA; 7 Department of Neurological Surgery, Northwestern University Feinberg School of Medicine, 303 East Superior St., Chicago, IL 60611 USA; 8 Department of Neurological Surgery, Wakayama Medical University, 811-1 Kimiidera, Wakayama, Japan; 9 Children’s National Medical Center, 111 Michigan Avenue NW, Washington, DC 20010 USA; 10 University Children’s Hospital Zurich, Steinwiesstrasse 75, 8032 Zurich, Switzerland; 11 Department of Radiation Oncology, New York University Langone Health, 550 First Avenue, New York, NY 10016 USA; 12 Department of Pathology, Robert H. Lurie Cancer Center, Northwestern University Feinberg School of Medicine, 303 E. Chicago Ave., Chicago, IL 60611 USA; 13 Department of Pharmacology and Toxicology, Michigan State University, 1355 Bogue St, East Lansing, MI 48824 USA; 14 Department of Computer Science and Engineering, Michigan State University, 428 S. Shaw Lane, East Lansing, MI 48824 USA; 15 Robert H. Lurie Comprehensive Cancer Center, Northwestern University Feinberg School of Medicine, 303 East Superior St., Chicago, IL 60611 USA

**Keywords:** Diffuse intrinsic pontine glioma, Computational approach, Machine learning, Drug repurposing, Mycophenolate mofetil

## Abstract

Diffuse intrinsic pontine glioma (DIPG) is an aggressive incurable brainstem tumor that targets young children. Complete resection is not possible, and chemotherapy and radiotherapy are currently only palliative. This study aimed to identify potential therapeutic agents using a computational pipeline to perform an in silico screen for novel drugs. We then tested the identified drugs against a panel of patient-derived DIPG cell lines. Using a systematic computational approach with publicly available databases of gene signature in DIPG patients and cancer cell lines treated with a library of clinically available drugs, we identified drug hits with the ability to reverse a DIPG gene signature to one that matches normal tissue background. The biological and molecular effects of drug treatment was analyzed by cell viability assay and RNA sequence. In vivo DIPG mouse model survival studies were also conducted. As a result, two of three identified drugs showed potency against the DIPG cell lines Triptolide and mycophenolate mofetil (MMF) demonstrated significant inhibition of cell viability in DIPG cell lines. Guanosine rescued reduced cell viability induced by MMF. In vivo, MMF treatment significantly inhibited tumor growth in subcutaneous xenograft mice models. In conclusion, we identified clinically available drugs with the ability to reverse DIPG gene signatures and anti-DIPG activity in vitro and in vivo. This novel approach can repurpose drugs and significantly decrease the cost and time normally required in drug discovery.

**Supplementary Information:**

The online version contains supplementary material available at 10.1186/s40478-022-01463-z.

## Introduction

Diffuse intrinsic pontine glioma (DIPG) is one of the highly aggressive pediatric gliomas that grows diffusely in the pons of the brainstem. It mostly affects children between 5 and 10 years of age, with a median survival of less than one year and a 99% 5-year mortality [27, 34]. Because of its unresectable tumor location and diffusive nature, and its resistance to conventional chemotherapy agents such as temozolomide, radiotherapy remains the standard treatment currently that has demonstrated clinical efficacy [17]. Yet DIPG still remains a fatal disease.

Molecularly, 80% of DIPG tumors harbor a lysine-to-methionine substitutions (K27M) in genes encoding histone H3 [30, 50, 60], which marked the first disease with known associations between histone mutations and cancer. These findings were made possible by the expanded studies of increased tumor biopsy and autopsy samples in the past decade, which also resulted in a growing DIPG gene expression dataset. Grasso et al. has used a chemical screen of patient-derived DIPG cultures along with RNA sequence (RNAseq) analysis to identify histone deacetylase (HDAC) inhibitor panobinostat, a Food and Drug Administration (FDA)-approved drug for the adult hematological malignancy multiple myeloma, as a potential therapeutics for the treatment of DIPG, as it restores H3-K27 methylation and subsequent normalization of gene expression [25].

Taking advantage of the increasing availability of large public dataset on disease-specific and drug-induced transcriptomic signatures, a computational approach to identify repurposed drugs for cancer treatment has been developed. This approach starts with computing a disease gene expression signature by comparing tumor samples with control samples, followed by identifying drugs that have a reversal relationship with the disease signature [12]. Using this method, we have identified repurposed drugs for the treatment of various cancers such as Ewing’s sarcoma and hepatocellular carcinoma, and successfully validated the drugs both in vitro and in vivo [12, 13, 39]. We further observed that the reversal of disease gene expression correlates with drug efficacy [12].

Unlike previous studies, in which counterpart normal control tissue samples were easily obtained and accessible, normal brain tissue samples were rarely obtained from the same patient. In this study, we applied a machine-learning-based drug-repurposing pipeline to identify drug candidates that can reverse the DIPG gene signature derived from an integrative analysis of bulk RNAseq and single-cell RNAseq (scRNAseq) datasets and therefore have the potential to treat the disease. Three drugs/compounds, which have not been studied in DIPG previously, were predicted, with mycophenolate mofetil (MMF), an immunosuppressive drug as a top hit. We evaluated the anti-tumor activities of the drugs in DIPG cell lines, and further validated MMF effects in DIPG mouse models.

## Materials and methods

### Data sets

We searched European Genome-phenome Archive (EGA) and Sequence Read Archive (SRA) using the key word “diffuse intrinsic pontine glioma” and identified one DIPG dataset (EGAS0000100192) provided by St. Jude Children’s Research Hospital in 2018 [61]. It included 22 DIPG (15 samples with H3K27M mutation) and 44 non-brainstem high-grade glioma raw RNAseq samples. Additionally, we acquired 28 raw DIPG RNAseq samples from Children's Brain Tumor Tissue Network (CBTN). These samples were integrated into the Open Cancer TherApeutic Discovery (OCTAD) database that included RNAseq data from 7412 specimens obtained from non-cancer donors. Neither of the DIPG datasets included matched healthy tissue. The RNAseq by Expectation Maximization (RSEM) data of the scRNAseq of 2259 malignant cells and 232 non-malignant oligodendrocyte cells were downloaded from the Broad Institute Single Cell Portal [20]. Drug sensitivity data in four DIPG cell lines (SU-DIPG-IV, JHH-DIPG-1, SU-DIPG-XIII, SU-DIPG-VI) were downloaded from NCATS Matrix [33]. Only the half maximal activity concentrations (AC50s) with CCLASS2 < 4 and CCLASS < 4 (strong signal) were kept, leaving 1326 compounds for the following comparison. Raw RNAseq data were processed using the pipeline adopted in OCTAD [63]. In addition, RUVg [49] (with 5000 empirically differentially expressed genes) was applied to remove unwanted variation, and weakly expressed genes were removed while computing differentially expressed genes. Normalized raw counts were used for differential expression (DE) analysis, and Transcripts Per Million (TPM) was used for other analyses. The clustering of these samples with DIPG samples compared to brain samples and other tissues demonstrates the feasibility of performing differential expression analysis between tissue samples.

### RNAseq processing

To minimize the batch effect from multiple studies, we used the same pipeline TOIL developed by University of California, Santa Cruz (UCSC) to process all raw RNAseq profiles [58]. STAR [20] was used for alignment and read coverage. RSEM [32] was employed to estimate transcript abundance. Because the UCSC Treehouse initiative has already used this pipeline to process samples publicly available (https://treehousegenomics.soe.ucsc.edu/public-data/), we decided to use their processed samples and extend this pipeline to process new samples (https://github.com/Bin-Chen-Lab/chenlab_toil). New samples from the major RNAseq repositories including GEO, dbGAP, and EBI EGA are routinely processed using this pipeline. Moreover, we further normalized the raw counts using RUVg (with 5000 empirically differentially expressed genes) [44] prior to differential gene expression analysis.

### Deep-learning based tissue selection

In many cancers including DIPG, adjacent normal tissues are not readily accessible. The Genotype-Tissue Expression (GTEx) project provides a rich RNA-seq repository for samples of healthy individuals; however, because their profiles were generated from different studies and processed under different computational approaches, the direct use of these profiles as a surrogate for adjacent normal tissues was largely unknown. Our previous study performed a systematic comparison of commonly used approaches for selecting normal tissue RNAseq from GTEx and proposed a deep learning autoencoder method to assist the selection of normal samples [64]. An autoencoder is an unsupervised deep learning method that learns the representation of input data with the goal of finding an optimal embedding. Compared with other dimensional deduction methods such as principal component analysis, an autoencoder can capture non-linear relationships between input features, thus presenting an unique advantage to embed gene expression features. Briefly, an autoencoder was trained using the entire OCTAD TPM matrix (including DIPG samples) with the following parameters: 64 encoded features, 128 batch size, 100 epochs, 0.0002 learning rate. Rectifying activation function, dropout and normalization were applied between layers. Afterwards, the embedded profiles of DIPG samples were compared to those from all GTEx normal samples. The top 100 highly correlated ones were selected as normal controls, as previously described [63, 64].

### Disease signature creation

To obtain corresponding tissues, we computed pairwise correlation between 22 DIPG samples and all 7412 healthy samples using the encoded gene expression features computed from a deep learning autoencoder [64]. Compared to conventional feature selection methods such as top varying genes and principal component analysis, deep learning autoencoder could capture non-linear correlations of the gene features, making it more appropriate to select precise reference tissues. Grouping by tissue of origin allowed to find the closest healthy tissues later to be used to compute disease signatures. The edgeR method wrapped in the OCTAD R package was employed to perform differential DE analysis (log2 fold change > 1, adjusted *p*-value < 0.05) [45, 63]. EnrichR wrapper was used for pathway enrichment analysis [14]. The R package ClusterProfiler was used for pathway enrichment analysis and visualization. The detailed data processing and parameter selection were detailed elsewhere [63]. The GSEA enrichment tool [54] was adapted for visualization. For scRNAseq analysis, the fast student’s t test implemented in the R package matrixTests was applied to log (RSEM + 0.1) to compute DE genes (log2 fold change > 1 and adjusted *p* vale < 0.05). Genes with variation across cells < 0.1 were removed in the following analysis. In addition, through literature curation, we compiled six published DIPG signatures from four studies: 1) Paugh_2011 (DIPG vs. high-grade glioma) [38], 2) Saratsis_2014 [DIPG vs. other brain tumors, DIPG vs. normal brains (Brainstem or Frontal Lobe)] [47], 3) Pathania_2017 (pediatric high-grade gliomas vs. normal brains) [37], 4) Anastas_2019 (KDM1A high vs. low, increased vs. decreased survival) [5].

### Drug prediction

The Library of Integrated Cellular Signatures (LINCS) database, including gene expression profiles for compound-treated cells, has been extensively used for drug prediction in a wide range of diseases including Alzheimer’s [56] and melanoma [35], in which the relevant cell lines are not even included in LINCS [35]. The library comprises 476,251 signatures and 22,268 genes including 978 landmark genes. The 1974 mapped drugs listed in the Repurposing Hub were considered here [18]. To compute reversal gene expression scores (RGES), which are a quantitative measurement of how well a compound reverses a gene signature, we first ranked genes based on their expression values in each drug profile and estimated the enrichment of up/down regulated disease genes in the ranked drug profile using the Kolmogorov–Smirnov-test. We chose the top 100 up or down landmark disease genes when the gene size exceeded 100. One compound might have multiple available expression profiles because they were tested in various cell lines, drug concentrations, treatment durations, or even different replicates, resulting in multiple RGES for one drug-disease prediction. We summarized multiple RGES as sRGES based on a simple statistics proposed before [12]. The computation of RGES and the summarization RGES were detailed and implemented as a standalone R package in our recent study [63]. A sRGES threshold of -0.1 was the cutoff for compounds which effectively reversed the disease signature.

### Cell sources and propagation

The primary human DIPG cell line SF8628 (H3.3K27M) and human glioblastoma (GBM) cell lines, SF9402 (H3 wild type), SF9427 (H3 wild type), and U-87 MG were obtained from the University of California, San Francisco (UCSF) medical center in accord with an institutionally approved protocol. Establishment of SF8628 from a surgical specimen and tumor cell modification for expression of firefly luciferase for in vivo bioluminescence imaging (BLI) has been described [6, 28, 29, 40, 43]. SU-DIPG-IV cell lines was kindly provided by Dr. Michelle Monje (Stanford University, Stanford, CA) CNMC-D-1428 suspension cell line was kindly provided by Dr. Javad Nazarian (Children’s National Hospital, Washington DC). Cell lie KNS-42, with H3.3G34V mutation (substitution of glycine 34 with valine), was obtained from Japanese Collection of Bioresources. Cells were maintained in a humidified incubator at 37℃ and 5% CO_2_. SF8628, KNS-42, and U-87 MG cells and normal human astrocytes (NHA) were grown in DMEM supplemented with 10% FBS and 1 × antibiotic-antimycotic. SU-DIPG-IV cells were maintained in tumor stem medium (TSM) which consisted of 50% of neurobasal-A medium, 50% of DMEM/F12 medium, 10 mM of HEPES buffer, 1 mM of MEM sodium pyruvate, 100 µM of MEM non-essential amino acids, 1 × GlutaMAX-I and 1 × antibiotic-antimycotics, supplemented with B27 minus vitamin A supplement, 20 ng/ml of hEGF and 20 ng/ml of hFGF. CNMC-D-1428 suspension cells were maintained in TSM medium plus hPDGF-AA and hPDGF-BB (10 µg/ml each). Cell lines were authenticated by short tandem repeat (STR) profiling, and routinely verified free of mycoplasma infection by the Venor GeM Mycoplasma Detection kit (Millipore-Sigma, St. Louis, MO, USA).

### Cell viability assay

Cells were seeded in a 96-well plate at a density of 1000–2000 cells per well and treated with various concentrations of triptolide (Sigma-Aldrich, St. Louis, MO, USA), triamterene (Sigma-Aldrich, St. Louis, MO, USA), MMF (Selleck Chemicals, Houston, USA), and mycophenolic acid (MPA, Sigma-Aldrich, St. Louis, MO, USA) for 3 or 6 days. Cell viability was measured using WST1 reagents (Takara, Kusatsu, Japan) at the end of the treatment. IC50s were determined by non-linear regression using GraphPad Prism 8.

### RNA extraction and RNA seq for treatment samples

SU-DIPG-IV and SF8628 cells were treated with appropriate drugs at their IC50 concentration for 24 h and RNA was extracted using the RNeasy plus mini kit (Qiagen, Hilden, Germany). RNA was then sent for sequencing by Novogene. The raw sequences were processed by the OCTAD pipeline. Differential gene expression was calculated using edgeR.

### Xenograft studies

Six-week-old female athymic mice (nu/nu genotype, BALB/c background) were purchased from Envigo (Indianapolis, IN, USA) and housed under aseptic conditions. For intracranial xenograft models, pontine injection of tumor cells was performed as previously described [8, 25, 28, 29, 45]. Each mouse was injected with 1 µL cell suspension (100,000 cells/µL) into the pontine tegmentum 4.5 mm deep from the inner base of the skull. Animals were randomly assigned to control vehicle [dimethyl sulfoxide (DMSO), n = 7] and MMF treatment groups [intraperitoneal (IP) injection of 50 mg/kg MMF for 15 days for 3 weeks, n = 9]. Mice with intracranial tumor began receiving the treatment on day 16 when consecutive BLI indicates a logarithmic tumor growth in all mice. Mice were monitored daily and euthanized at the endpoint, which included irreversible neurological deficit or a body condition score less than 2. For convection-enhanced delivery (CED), 1% of DMSO (n = 7) or 1 mM of MMF (n = 7) in 5% sucrose with a volume of 10uL was directly infused to the intracranial tumor at a rate of 1 uL/min for 10 min using micro-infusion pump as previously described [48, 51]. For subcutaneous xenograft models, SF8628 cells were implanted into the flank of athymic mice as previously described [26]. Briefly, 4 × 10^6^ cells, in 0.4 ml of cell culture media with matrigel (BD Bioscience) at 1:1 ratio, were injected in the right flank of mice under anesthetization by isoflurane. Mice were randomly assigned to vehicle (DMSO, n = 7) and MMF treatment (IP of 100 mg/kg for 15 days for 3 weeks, n = 7) groups when the size of tumor reached at 100 mm3. The tumor sizes were measured twice a week and the mice were euthanized when the tumor size reached 1000 mm^3^. All protocols, described below, were approved by the Northwestern University Institutional Animal Care and Use Committee.

### Analysis of drug concentration in the brain

Athymic mice were administered 50 mg/kg of MMF for 5 days, with brains resected and serum collected following mouse euthanasia at one hour after the fifth administration (n = 2). The brainstem was dissected from the surrounding brain, the serum was collected by cardiac puncture, and the samples were snap frozen and stored at − 80 °C. MMF was extracted from homogenized tissues using a Bullet Blender (Next Advance, Troy, NY, USA). Homogenates were extracted with organic solvent and were transferred to an autosampler for liquid chromatography–mass spectrometry (LC/MS) analysis (VP Series 10 System; Shimadzu, Kyoto, Japan) for determination of MMF content (Integrated Analytical Solutions, Inc, Berkeley, CA, USA). Brain penetration ratio was calculated as MMF brainstem concentration divided by serum concentration (Table 1).

### Immunohistochemistry (IHC)

Brains were collected from the mice 3 h after completion of the last treatment (n = 2 for each treatment). Paraformaldehyde-fixed brains were paraffin-embedded and sectioned (10 µm) for hematoxylin and eosin (HE) and anti-Ki67 antibody (2 µg/mL) (Ventana, Tucson, AZ, USA) staining. To assay the apoptotic response to treatment, TUNEL staining was performed using the DeadEnd Colorimetric TUNEL system (Promega, Madison, WI, USA) according to the manufacturer’s protocol for paraffin-embedded tissues.

### Statistical analysis

For in vivo study, the Kaplan–Meier estimator and Prism software were used to generate and analyze survival plots. Differences between survival plots were calculated using a log-rank test. A 2-tailed unpaired *t*-test was used (GraphPad Software, San Diego, CA, USA) for comparison the tumor size between each treatment group.

## Results

### DIPG disease gene signature and drug hit predictions

**Fig. 1:**
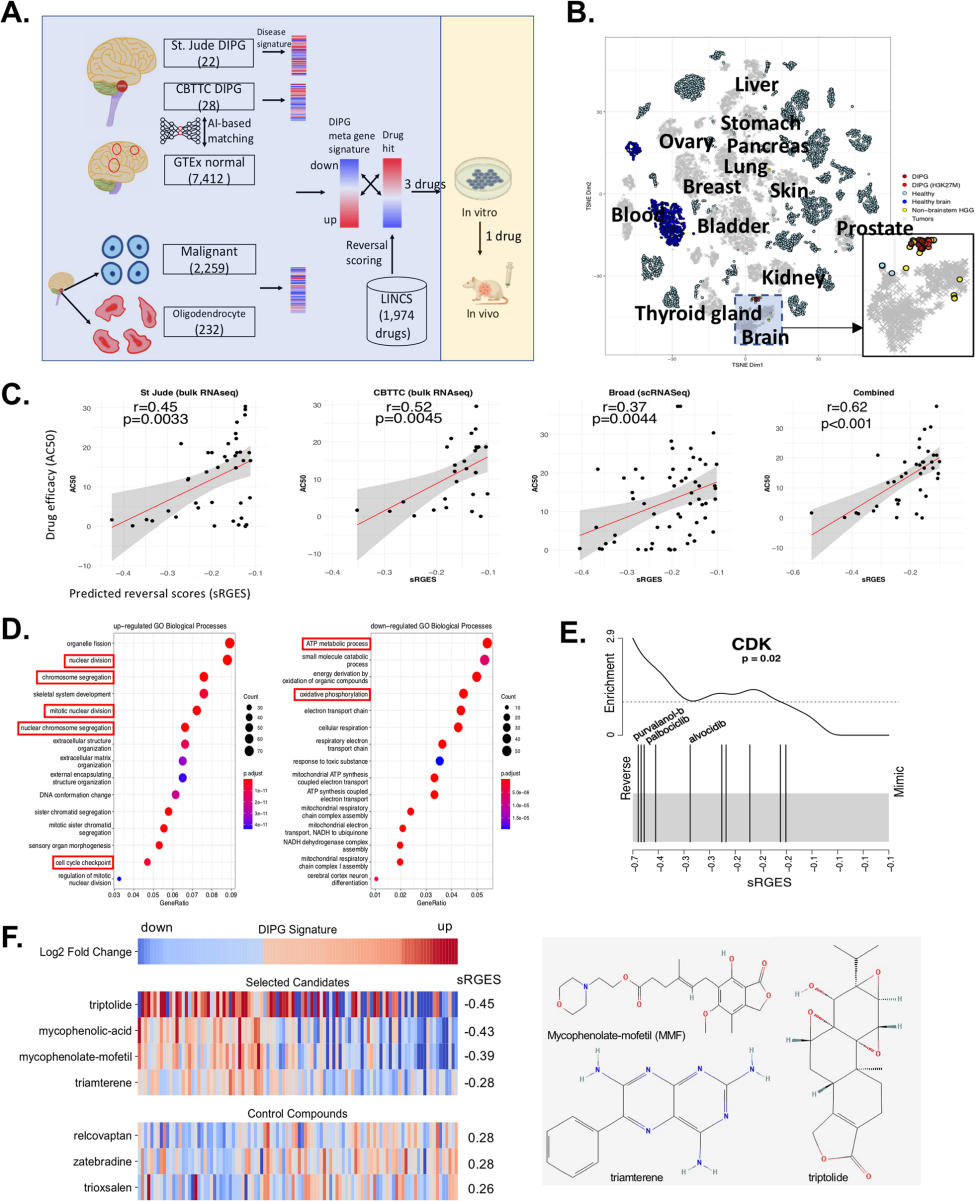
Prediction of DIPG drug hits. **A** Working model. In vitro/in vivo illustrations were taken from Biorender. **B** DIPG samples in a tumor map comprising > 17,000 samples. The tumor map is a t-distributed stochastic neighbor embedding (t-SNE) plot of sample TPM expressions. DIPG and brain cancer samples are highlighted. **C** Correlation between predicted reversal gene expression scores (sRGES) and experimental drug efficacy with a half maximal activity concentration (AC50) value. A lower sRGES means a higher potency to reverse the DIPG signature genes. The drug corresponding to the dot plot are described in Additional file 1: Table S1). **D** Meta-disease genes and their enriched pathways. The pathways commonly changed by the predicted drugs are highlighted. **E** Enriched target class of CDK in the predictions. The black line on the left suggests it is ranked on the top in the prediction list. An enriched target class means the ligands of the target tend to be highly ranked in the predictions. The enrichment of other two targets (TOP, HDAC) are illustrated in Additional file 4: Fig. S1(three eample drugs are labeled in each plot). **F** Top candidates selected for validation. The first row shows the meta disease signature. The signatures of four top candidates and three randomly selected control compounds are visualized. The altered genes are listed in Additional file 6: Table S5

Like many brain cancers, the matched biopsies are difficult to acquire in DIPG. Neither DIPG cohorts provided RNAseq of matched tissues, thus creating a disease signature is not possible using the existing pipeline. GTEx built up a large RNAseq repository for healthy individuals, providing an opportunity to use GTEx as a surrogate (Fig. 1A). Using the same RNAseq processing pipeline to harmonize the publicly available tumor samples and normal tissue samples resulted in a tumor map consisting of 19,127 samples, in which DIPG samples cluster together beside non-brainstem glioma samples (Fig. 1B). We further observed that DIPG samples with H3K27M mutation do not separate from the rest, suggesting the shared transcriptomic features among DIPG samples. Even though all the raw sequences were processed under the same pipeline, the batch effect may remain. This emerged problem motivated us to develop a deep learning autoencoder that normalizes and compresses gene expression profiles into a smaller feature space that allows the selection of appropriate reference control samples from GTEx. Using this approach, we selected 100 healthy RNAseq tissues mostly correlated to DIPG samples from the St Jude Children’s Research Hospital, the dataset we first acquired, normalized with DIPG samples, and then created a disease signature comprising 1859 up and 2990 down regulated genes.

We next applied the disease signature and computed the reversal score of the drugs using the OCTAD pipeline. We chose the compounds with lower sRGES (sRGES < − 0.1, lower sRGES suggests higher reversal potency) and with high-quality experimental drug sensitivity data (measured by AC50, a lower AC50 means higher efficacy). We observed a positive significant correlation between reversal potency and drug sensitivity (Pearson R = 0.45, *P* < 0.01) (Fig. 1C, Additional file 1: Table S1), similar to what we observed in other cancers [12], implying that the drugs that present high reversal potency, yet experimentally tested could be effective. Tuning the parameters to select reference controls indeed changed the correlation, but the variation was subtle. Later, we acquired another DIPG dataset from CBTN, and performed a similar analysis. Both disease signatures resulted in a significant correlation between predictive reversal potency and drug sensitivity (R = 0.52, *P* < 0.01) (Fig. 1C); however, combining both signatures led to a much high correlation (R = 0.62, *P* < 0.01) (Fig. 1C), suggesting a robust signature after the combination. Gene Ontology (GO) enrichment analyses revealed mitotic cell cycle, chromosome segregation, and glycosaminoglycan metabolic process were up-regulated, and oxidative phosphorylation (OXPHOS), mitochondrial electron transport related pathways and cellular response to zinc ion were down-regulated (Fig. 1D, red squre).

In parallel to the bulk RNAseq, scRNAseq reached a momentum in the past few years. Filbin et al*.* released a SMART-Seq based scRNAseq of six DIPG patients [22]. The comparison of malignant cells and oligodendrocytes resulted in a signature of 3443 genes, but it did not lead to a significant correlation between predicted reversal potency and the experimental data. This is likely because of cell heterogeneity, thus we performed a similar analysis using the malignant cells from each of the four programs (Cell cycle, OPC like, AC like and OC like) with distinct transcriptomic features. Only the signature derived from the cell cycle program reached a very significant correlation. However, combining the scRNAseq signature and bulk RNAseq signature did not improve the performance (R = 0.62, *P* < 0.01, Fig. 1C). We further assessed six published DIPG gene signatures using the identical evaluation matrix. None of them are superior, likely because they were not developed or optimized to support drug screening (Additional file 2:Table S2). Therefore, we used a meta-signature derived from the two bulk RNAseq datasets to run the drug prediction (Additional file 3: Table S3).

The drug-target analysis of drug hits suggested the enrichment of known drug classes including CDK inhibitors (Fig. 1E), Topoisomerase (TOP) inhibitors, and HDAC inhibitors (Additional file 4: Fig. S1), justifying the prediction results. We then excluded known or non-specific chemotherapy drugs (TOP inhibitors, CDK inhibitors, HDAC inhibitors, DNA inhibitors) in order to identify new clases of drugs for DIPG therapy. We further filtered out the drugs with less than three profiles (lower confidence due to limited samples), and preclinical drugs, leaving triptolide as the top hit, followed by MPA and MMF (Top 20 hits available in Additional file 5: Table S4). The following seven hits include dabrafenib, clofarabine, methotrexate, loteprednol, actinomycin-d, idoxuridine, and triamterene. Some of them are known anti-cancer drugs such as dabrafenib, clofarabine, methotrexate, and actinomycin. Notably both MMF and MPA had > 10 drug profiles and coincidently were ranked among top three (Fig. 1F, Additional file 6: Table S5). We next chose triptolide, MMF, and triamterene for the following experimental validation.

### Predicted drugs decreased cell viability in DIPG cell lines

**Fig. 2:**
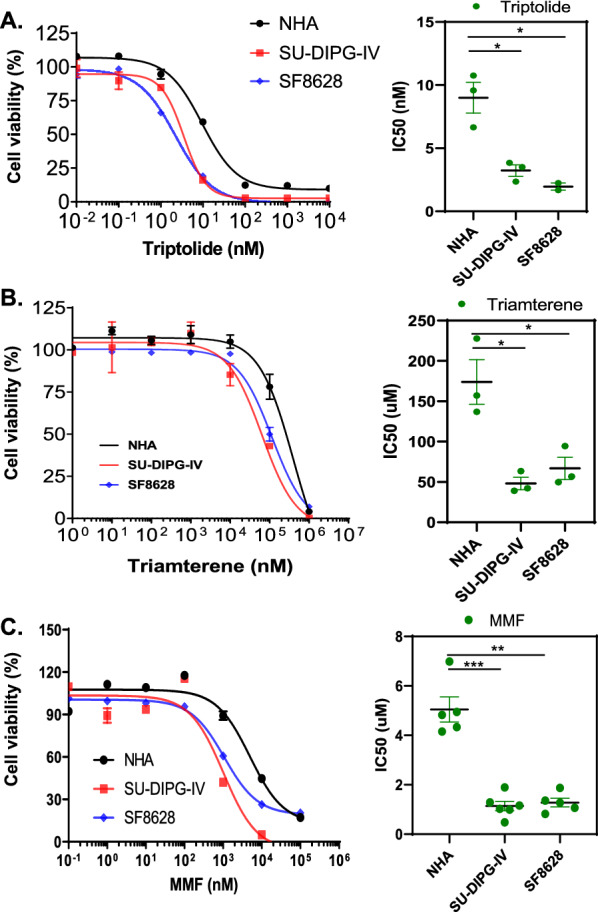
Cell viability assay valuation of the predicted drugs. Triptolide (**A**), triamterene (**B**) and mycophenolate mofetil (**C**) in DIPG cell lines (SF8628, SU-DIPG-IV) as well as control NHA cells. Left: Graphs showing the proliferation response of normal human astrocytes (NHA) and DIPG cell lines (SU-DIPG-IV, SF8628), to increasing concentrations of each drug. Values shown are the average [mean ± standard deviation (SD)] from triplicate samples for each incubation condition. Right: Dot plot representation of IC50 values shown are the average (mean ± SD) from triplicate samples for each cell lines. Statistical analysis was performed using a two-tailed unpaired t-test: triptolide, NHA versus SU-DIPG-IV, *P < 0.05; NHA versus SF8628, *P = 0.05; triamterene, NHA versus SU-DIPG-IV, *P < 0.05; NHA versus SF8628, *P < 0.05; MMF, NHA versus SU-DIPG-IV, ***P < 0.001; NHA versus SF8628, **P < 0.01

We validated the efficacy of predicted drugs by measuring cell viability in two DIPG cell lines SU-DIPG-IV and SF8628. Comparing to NHA cells, all 3 drugs decreased cell viability in DIPG cell lines (Fig. 2). Triptolide showed the highest efficacy with IC50s in 2–3 nM concentration (Fig. 2A). Triamterene showed efficacy in treating DIPG cells compared to NHA but required a large dose (more than 50 µM) to achieve the treatment effect (Fig. 2B). MMF, an FDA-approved immunosuppressive drug commonly used in organ transplant, showed high efficacy in decreasing cell viability in DIPG cells (Fig. 2C), with an IC50 value less than that of NHA cells (NHA: 4.94 µM, SU-DIPG-IV: 1.02 µM, SF8628: 1.06 µM). Comparing to non-H3K27M mutant glioma cells, MMF showed greater inhibition on cell growth of H3K27M mutant DIPG cells (SU-DIPG-IV, SF8628) than that observed in H3 wild type (SF9427, SF9402), H3G34V mutant (KNS-42), or IDH wild type (U-87 MG) GBM cell lines (Additional file 4: Fig. S2).

### RNAseq data showed MMF can reverse gene signature in DIPG cell lines

**Fig. 3:**
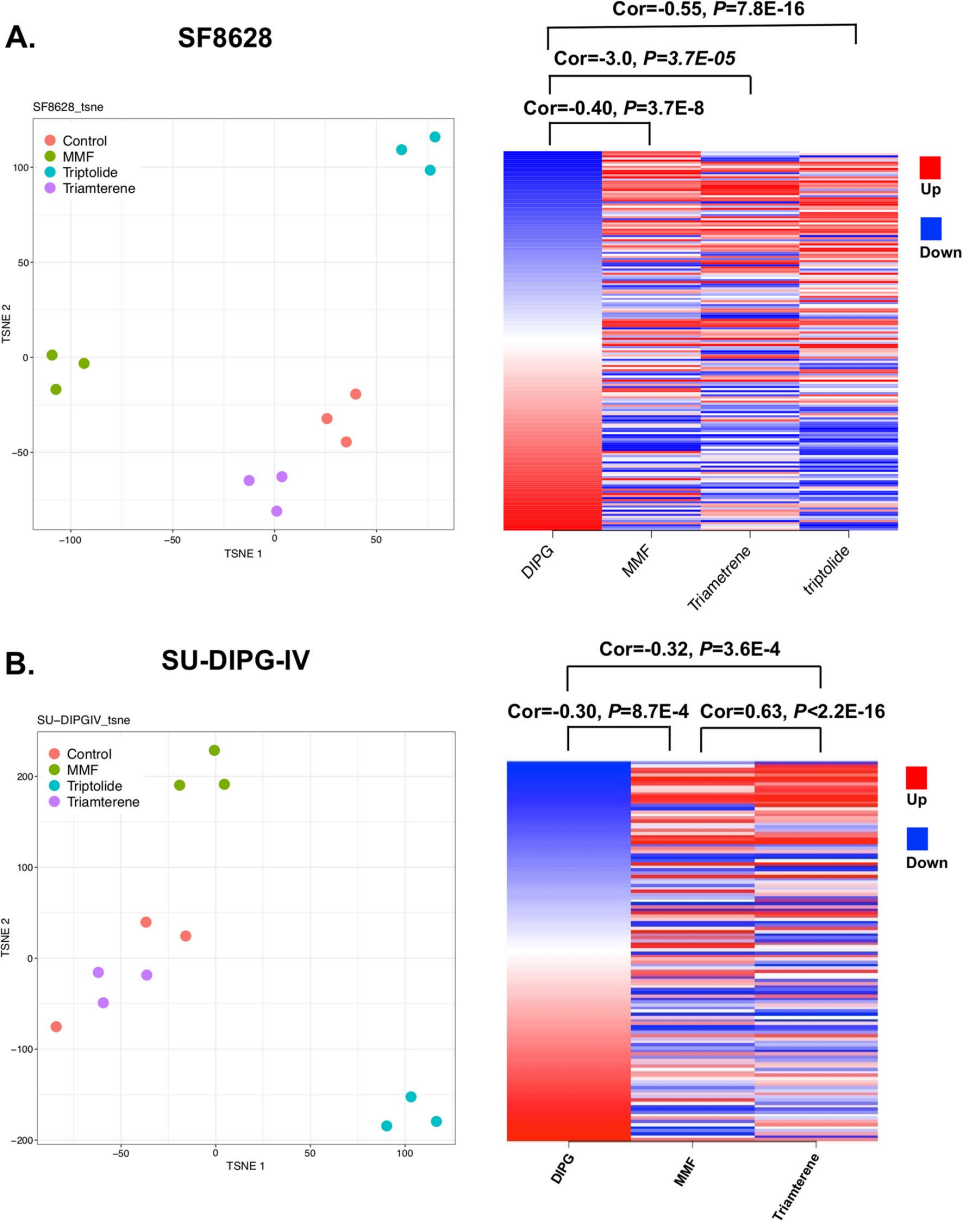
RNAseq analysis of treated samples in DIPG cells. t-SNE plot of treatment samples with vehicle control (0.1% DMSO), 1 µM of MMF, 2 nM of triptolide, and 30 µM of triamterene in SF8628 (**A**, left) and SU-DIPG-IV (**B**, left) cells. MMF reversed the DIPG disease gene expression in SF8628 (**A**, right) and SU-DIPG-IV (**B**, right) cells

H3K27M mutant DIPG cells (SF8628, SU-DIPG-IV) were treated with 1 µM of MMF, 30 µM of triamterene, and 2 nM of triptolide for 24 h and RNA was extracted and subjected for RNAseq. MMF, triamterene, and triptolide showed drug perturbation of gene expression in SF8628 cells (Fig. 3A, left). Triptolide, however, did not perturb SU-DIPG-IV cell gene expression as it clustered with vehicle-treated SU-DIPG-IV cells (Fig. 3B, left). MMF, triamterene, and/or triptolide treatment significantly reversed the gene signature in DIPG cell lines (Fig. 3A, right, Additional file 7: Table S6, Fig. 3B, right, Additional file 8: Table S7, Addiditional file 4: Fig. S3).

### MMF and MPA had similar efficacy in DIPG cells

**Fig. 4:**
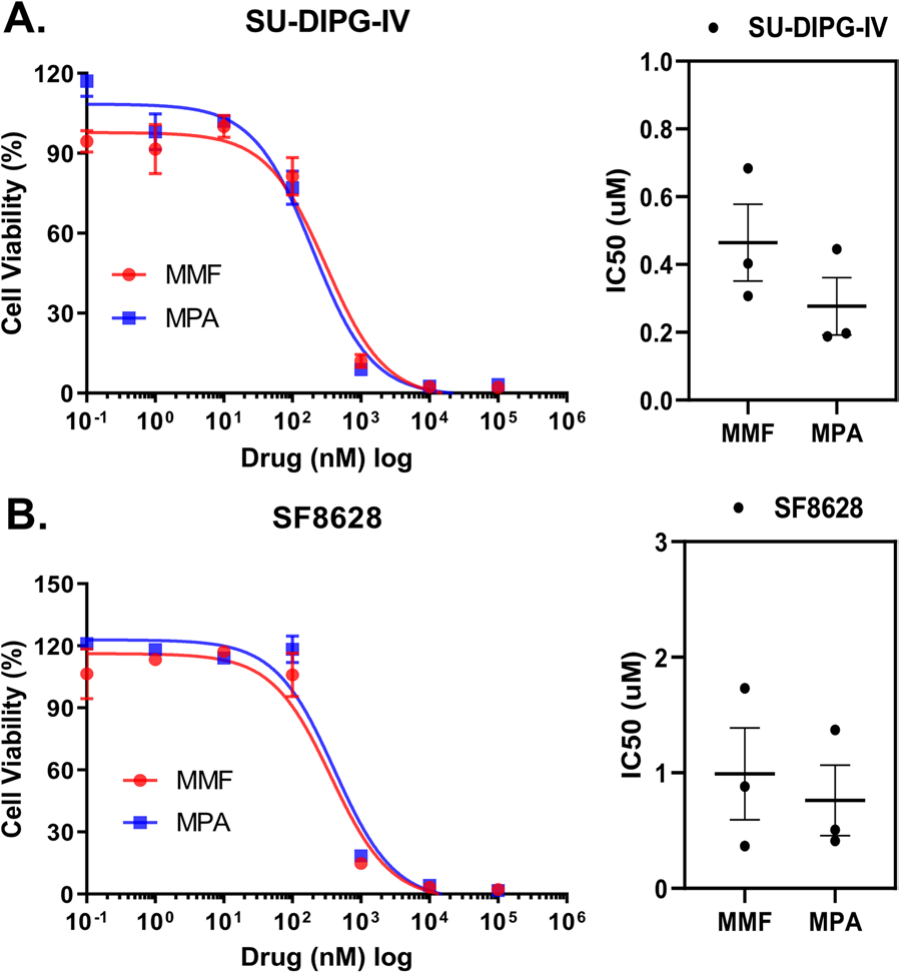
Comparison of the effects of mycophenolate mofetil (MMF) and mycophenolic acid (MPA) in DIPG cells. **A** Left: Graphs showing the proliferation response of SU-DIPG-IV, to increasing concentrations of MMF and MPA for 3 days treatment. Values shown are the average (mean ± SD) from triplicate samples for each incubation condition. Right: Dot plot representation of IC50 values shown are the average (mean ± SD) from triplicate samples for each treatment condition. **B** Left: Graphs showing the proliferation response of SF8628, to increasing concentrations of MMF and MPA treatment. Values shown are the average (mean ± SD) from triplicate samples for each incubation condition. Right: Dot plot representation of IC50 values shown are the average (mean ± SD) from triplicate samples for each treatment condition. Statistical analysis was performed using a two-tailed unpaired t-test. No significant differences were found among MMF and MPA

Since triptolide did not perturb SU-DIPG-IV gene expression (Fig. 3B), and a large dose was required for triamterene, we focused on MMF for further analysis. MMF is a prodrug that can be hydrolyzed to MPA. We compared the effects of MMF and MPA in H3K27M mutant DIPG cells. Both MMF and MPA induced a dose dependent growth inhibition of SU-DIPG-IV and SF8628 cells (Fig. 4).

### Guanosine can rescue decreased cell viability by MMF

**Fig. 5:**
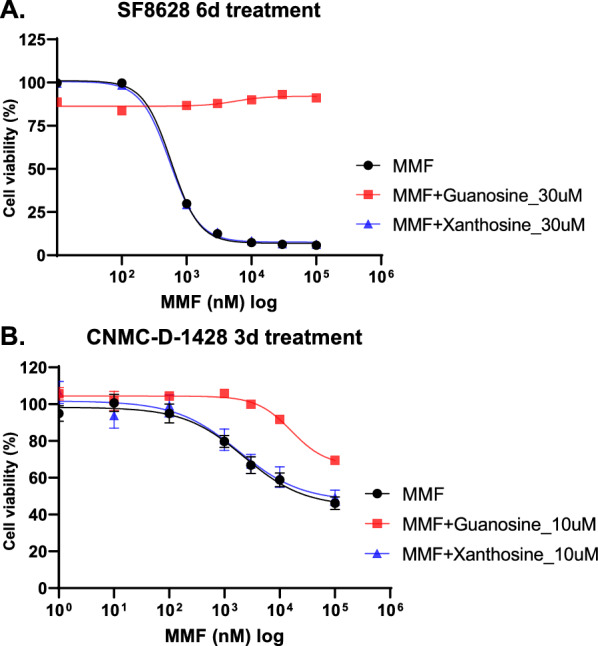
Guanosine but not xanthosine rescued MMF inhibitor response of DIPG cells. Graphs showing the proliferation response of SF8628 (**A**) and CNMC-D-1428 (**B**), to increasing concentrations of MMF in the presence of Guanosine or xanthosine treatment for 6 days in SF8628 cells or 3 days in CNMC-D-1428 cells. Values shown are the average (mean ± SD) from triplicate samples for each incubation condition

As MPA is an inhibitor of inosine-5’-monophosphate dehydrogenase 1 and 2 (IMPDH1 and IMPDH2) in de novo synthesis of guanosine nucleotides [2], we tested whether MMF exerts its effect in DIPG cells through the same mechanism. Of note, IMPDH2 was highly expressed in H3K27M mutant DIPG cell lines, when compared to NHA or H3-WT GBM cell lines (Additional file 4: Fig. S4). Guanosine was added to SF8628 cells (30 µM) or CNMC-D-1428 cells (10 µM) along with MMF treatment. As shown in Fig. 5, guanosine completely (in SF8628 cells) or partially (in CNMC-D-1428 cells) rescued the decreased cell viability, suggesting MMF inhibited DIPG cells by depleting guanosine nucleotides.

### MMF significantly increased overall survival in DIPG xenograft mouse models

**Fig. 6:**
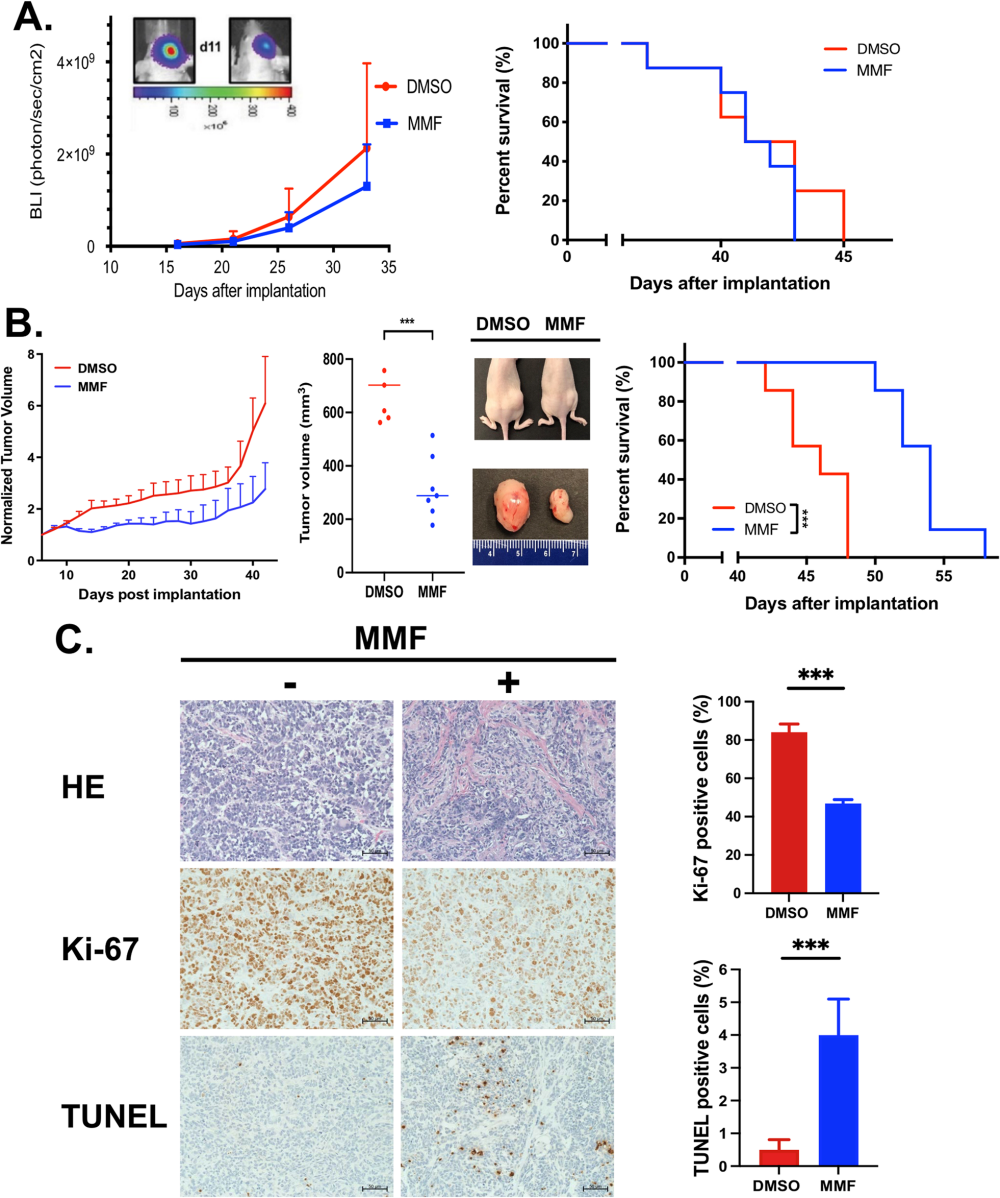
In vivo anti-tumor activity of MMF in patient-derived DIPG xenografted models. **A** Mice with SF8628 intracranial tumors were either treated with vehicle (DMSO, n = 7) or MMF (50 mg/kg for 15 days, n = 9). Left: Tumor growth curve for bioluminescence values in each treatment group. Tumor bioluminescence values show mean and upper SD. Upper left: Corresponding tumor bioluminescence intensity overlay images for representative DMSO (left) and MMF (right)-treated mice on day 11 post-tumor cell implantation. Right: Corresponding survival plots of each treatment group. **B** Mice with SF8628 subcutaneous (sc) tumor were either treated with vehicle (DMSO, n = 7) or MMF (100 mg/kg, n = 7) daily for 15 days for 3 weeks. Left: Growth plots for sc tumors in each treatment group. Tumor volumes were normalized against tumor volume obtained at day 6 post-tumor cell injection. Normalized tumor volume show mean and upper SD. Middle: Dot plot representation of sc tumor volume in mice at day 42 post-tumor cell injection. Unpaired *t*-test values for comparisons between DMSO and MMF treatment: ***P = 0.0002. Photographs of nude mice (upper) and sc tumor taken from these mice (lower) in which SF8628 cells were inoculated into the right flank. Right: Animal survival at the indicated days after inoculation. Log-rank test was used for comparisons between DMSO and MMF treatment: ***P = 0.0003. **C** Left: Images of representative Ki-67 and TUNEL staining for sc tumors from mice euthanized at the end of treatment. The scale bar is defined as the length of 50 µm. Right, mean and SD values representing the average number in positive cells in four-high-powered fields in each tumor. Statistical analysis was performed using the unpaired t-test. Ki-67: DMSO versus MMF, ***P = 0.0002. TUNEL: DMSO versus MMF, **P = 0.0060

Based on the biological effects of MMF in vitro, we hypothesized that MMF treatment suppresses tumor growth and increase survival in mice with orthotopic patient-derived DIPG xenografts. To determine the anti-tumor activity of MMF, the mice were implanted with SF8628 cells in the pons and were treated with 50 mg/kg of MMF for 15 days. MMF treatment delayed tumor growth while the treatment did not show survival benefit in the mice bearing intracranial (brainstem) DIPG xenografts (Fig. 6A). This could be due to poor permeability of MMF crossing the blood–brain barrier. To address the brain distribution of MMF compounds, we performed LC/MS analysis in the mice that were euthanized one hour following MMF administration. Their brains were immediately resected, the brainstem was dissected from the surrounding brain, and the serum was collected by cardiac puncture. LC/MS analysis of tissue extracts revealed an MMF concentration in the mice brainstem at 243.50 ± 50.20 ng/ml, which is only 1.07 ± 0.46% of serum concentration (Table 1).

**Table 1:** MMF concentration in brain and serum

*MMF concentration (ng/ml)*	
Brainstem	243.50 ± 50.20
Serum	26,150.00 ± 16,051.32
*MMF brain penetration ratio (%)*	
Brainstem / Serum	1.0747 ± 0.4677

Due to low brain penetration of MMF, we developed subcutaneous (sc) xenograft models to evaluate the MMF anti-tumor activity. We subcutaneously implanted SF8628 cells into the right flank of mice and treated the mice with MMF (100 mg/Kg) or vehicle intraperitoneally when tumor size reached at size of 100 mm^3^. Mice were euthanized when the tumor size reached to 1,000 mm^3^. MMF treatment significantly inhibited the sc tumor growth (*P* = 0.0002, Fig. 6B) and extend the survival of recipient mice with SF8628 sc xenografts compared to the control (DMSO treatment) group (*P* = 0.0003, Fig. 6B). This in vivo efficacy study included euthanizing the mice at the end of treatment to obtain sc tumor samples to analyze tumor cell proliferation (Ki-67) and apoptosis (TUNEL). IHC analysis of Ki-67 staining revealed MMF treatment significantly reduced Ki-67 positive cells (46.80 ± 1.68%) relative to DMSO treatment (84.10 ± 3.44%) (*P* = 0.0002, Fig. 6C). TUNEL staining results showed a higher proportion of positive cells in tumors from mice receiving MMF (4.00 ± 0.90%) relative to DMSO control (0.50 ± 0.25%) (*P* = 0.0060, Fig. 6C).

Alternative to systemic delivery of MMF, we treated orthotopic (brainstem) DIPG xenografts using a local drug delivery system, convection-enhanced delivery (CED). 10uL of 1 mM MMF was directly infused to the brainstem tumor using a mini-infusion pump as previously described [48, 51]. CED of MMF inhibited brainstem tumor growth and significantly extend survival of mice bearing SF8628 orthotopic xenopgrafts (*P* = 0.0439, Additional file 4: Fig. S5).

## Discussion

Drug repurposing offers a relatively short approval process and straightforward path to clinical translations. This study demonstrated a deep learning autoencoder for identifying reference controls from other studies, and combining disease signatures from two bulk RNAseq samples, resulted in the best predictive power analysis. The inferior performance of scRNAseq data might be due to the sparsity of scRNAseq and/or the suitability of using oligodendrocytes as a control. As more and more scRNAseq data becomes available, the comparable performance of scRNAseq-based signature could unleash the potential of applying scRNAseq in computational drug repurposing, in particular for individual patients.

In this study, we focused on the identification of new classes of drugs and excluded kown or non-specific chemotherapy agents (HDACs inhibitors, CDK inhibiors, TOP inhibitors) which have been investigated in DIPG. Lin et al. have used a high-throughput chemical screening of patient-derived DIPG cultures along with RNAseq analysis to identify HDAC inhibitor panobinostat, as a potential therapeutic agent for DIPG [33]. CDK inhibitors have been evaluated as single agents in multiple studies [9, 19]. TOP inhibitors have the potential to exacerbate DNA damage causing a high level of stress to the cancer cell [49]. This class of agents could be used in combination with radiation, which is currently used for DIPG patients. However, none of these chemotherapeutic strategy has been shown to improve overall survival in children with DIPG [41].

MMF is a prodrug of MPA, which is an inhibitor of IMPDH, a rate-limiting enzyme in de novo purine synthesis of guanosine nucleotides. MPA is an inhibitor of the type 2 isoform of IMPDH, which is expressed in activated T and B lymphocytes, and five-fold more potent than of the type 1 isoform, which is expressed in most cell types [11]. Because of the requirement of de novo purine synthesis in T and B lymphocytes proliferation [4], MMF was developed as an immunosuppressive drug [1]. With our machine-learning aided drug repurposing pipeline, we identified MMF as one of the top hits that can reverse the DIPG disease gene signature (Fig. 1F, Additional file 3: Table S3).

Since the drug reversal scores were computed based on the drug-induced gene expression profiles generated in non-DIPG cell lines, we generated new profiles in DIPG cell lines. We observed that MMF, triamterene, and/or triptolide treatment significantly reversed the DIPG gene expression (Fig. 3, Additional file 4: Fig. S3), suggesting the feasibility of using the computational approach to predict drugs for DIPG. Anti-cancer effect of MMF has been reported in various types of cancers [10], while immunosuppressive microenvironment is associated with progression, recurrence or metastasis of cancers [42]. Although combination of immunosuppressive drugs and anti-cancer drugs is not harmful in all cancers, for example combination of procarbazine and vincristine, an alkylating agent and an microtubule inhibitor, in addition to methotrexate, an immunosuppressive drug, is effective for primary central nervous lymphomas [36], further investigation would be required whether combination of MMF and antic-ancer drugs clinically used is effective or not in H3K27M-mutant gliomas.

As proliferating cancer cells have increased metabolic demands, biosynthesis of key molecules such as purines are of vital importance. Guanosine nucleosides, in particular, seem to be in high demand. Traut reported that GTP levels were 200% increased in cacner cells compared to normal cells, whereas ATP levels were only 20% increased [57]. In the analysis of prostate cancer cell, guanosine monophosphate (GMP) utilizes the metabolism of glutamine which is a critical nutrient in cancer [59]. Guanosine metabolism has been reported as a potential therapeutic target in other types of malignancy such as multiple myeloma [55], pancreatic cancer [46], prostate cancer [7, 59], hepatocellular carcinoma [16] or glioblastoma [31, 52] as well as our study. The anti-tumor effect of MMF in DIPG results from its inhibition of GMP synthesis, as exogenous GMP or guanosine can rescue the decreased cell viability caused by MMF in vitro (Fig. 5). However, exogenous xanthosine did not improve cell viability. Since no kinase activity exists for guanosine in human cells thus preventing its direct phosphorylation to GMP [53], the rescue effect of guanosine suggests that the salvage pathway of purine synthesis catalyzed by hypoxanthine-guanine phosphoribosyltrasnferase (HGPRT) is compensating for the de novo purine synthesis in DIPG cells. Xanthosine, on the other hand, does not appear to be phosphorylated by any ATP-mediated nucleoside kinases nor to be salvaged by the HGPRT pathway [23]. However, it was reported that xanthosine was salvaged to xanthosine monophosphate (XMP) via the phosphotransferase activity of cytosolic 5’-nucleotidase [8] in rat brain cytosolic extract and in intact human colorectal cancer cell line. It is possible that MMF/MPA can also inhibit GMP synthase, the enzyme catalyzing the conversion of XMP to GMP. Yet this function of MPA remains controversial [3].

In our in vivo efficacy study, MMF showed anti-tumor activity in the mice bearing sc xenografts (Fig. 6B). This is consistent with the results from the study using U-87MG GBM sc xenograft models [31]. However, MMF treatment did not show the survival benefit in the mice bearing intracranial (brainstem) DIPG xenografts (Fig. 6A) due to the low MMF brainstem concentration at 1.07 ± 0.46% of serum concentration (Table 1). Shireman et al. showed MMF treatment increase sensitivity of GBM intracranial xenogrfats to temozolomide, while MMF monotherapy did not show anti-tumor activity in vivo [52]. Additionally, Zhou et al. [65] demonstrated that purine synthesis regulates DNA repair in response to radiation in GBM models. MMF was found to significantly improve the anti-tumor efficacy of radiation both in vitro and in vivo. While the orthotopic GBM xenografts in this studies did show improved survival with the combination of orally administered MMF and radiation therapy, there was no efficacy of MMF alone, suggesting the possibility that the irradiation disrupted the blood–brain barrier thus improving drug penetration in those mice. Based on these findings, a clinical trial in adult GBM patients combining MMF with radiation is underway (ClinicalTrials.Gov: NCT04477200).

In this study as well as the previous studies from other groups [31, 52], MMF was administered systemically at daily dose of 100 mg/kg, which is approximately five times higher than recommend dose for use as an immunosuppressive agent in pedaitric patients [15, 21]. While the high dose are needed to achieve therapeutic levels, nonspecific distribution to the normal healthy tissue includung kidney can lead to substantial toxicity by systemic drug administration [24, 62]. In this study, we demonstarated the anti-tumor activity of CED of MMF in orthtotopic (brainstem) xenograft models (Additional file 4: Fig. S5), as an alternative strategy for direct drug delivery to the brainstem tumor. CED of MMF would reduce systemic toxicity and increase the therapeutic efficacy in vivo*,* and has a potential for next level clinical use.

## Conclusion

We identified clinically available drugs, MMF and MPA, with the ability to reverse gene signatures and anti-tumor activity for DIPG cell lines in vitro and in vivo. This novel approach can repurpose drugs and significantly decrease the cost and time normally required in drug discovery.

## Supplementary Information

**Additional file 1: Table S1**. The list of drugs with predicted reversal predicted reversal gene expression scores (sRGES) and experimental drug efficacy (AC50).

**Additional file 2: Table S2**. Comparison of gene signature of DIPG in six previously published data.

**Additional file 3: Table S3**. Meta-signature derived from the two bulk RNAseq datasets.

**Additional file 4:  Supplementary Figures S1**. Enriched target class of TOP (top) and HDAC (bottom) in the predictions.** Supplementary Figures S2**. Cell viability assay valuation of MMF. **Supplementary Figures S3**. RNAseq analysis of MMF-treated SF8628 (A) and SU-DIPG- IV (B) cells. **Supplementary Figures S4**. Expression of IMPDH2 in pediatric high-grade glioma cells. **Supplementary Figures S5**. In vivo anti-tumor activity of MMF by CED in orthotopic patient-derived DIPG xenografts.

**Additional file 5: Table S4**. Top 20 hits of drugs identified by machine-learning aided drug repurposing pipeline in this study.

**Additional file 6: Table S5**. The list of altered genes with corresponding pathways.

**Additional file 7: Table S6**. The list of differential expression genes upon drug treament in SF8628 cells.

**Additional file 8: Table S7**. The list of differential expression genes upon drug treament in SU-DIPG-IV cells.

## Data Availability

Raw RNASeq reads and processed HTSeq read counts are available on GEO under GEO GSE193855 (token ipkzqwgezjarzsf). The main code is available at https://github.com/Bin-Chen-Lab/dipg. The other datasets and brain tumor models used and/or analysed and during the current study available from the corresponding author on reasonable request.

## Abbreviations

AC50: A half maximal activity concentration
BLI: Bioluminescence imaging
CBTN: Children's brain tumor tissue network
CED: Convection-enhanced delivery
DE: Differential expression
DIPG: Diffuse intrinsic pontine glioma
DMSO: Dimethyl sulfoxide
EGA: European genome-phenome archive
FDA: Food and drug administration
GBM: Glioblastoma
GMP: Guanosine monophosphate
GO: Gene ontology
GTEx: Genotype-tissue expression
HDAC: Histone deacetylase
HE: Hematoxylin and eosin
HGPRT: Hypoxanthine-guanine phosphoribosyltrasnferase
IMPDH: Inosine-5’-monophosphate dehydrogenase
IP: Intraperitoneal
K27M: A lysine-to-methionine substitutions
LC/MS: Liquid chromatography–mass spectrometry
LINCS: Library of integrated cellular signatures
MMF: Mycophenolate mofetil
MPA: Mycophenolic acid
NHA: Normal human astrocytes
OCTAD: Open cancer therapeutic discovery
OXPHOS: Oxidative phosphorylation
RSEM: RNAseq by expectation maximization
RGES: Reversal gene expression scores
RNAseq: RNA sequencing
sc: Subcutaneous
scRNAseq: Single-cell RNA sequencing
SD: Standard deviation
SRA: Sequence read archive
sRGES: Summarized RGES
STR: Short tandem repeat
TOP: Topoisomerase
TPM: Transcripts per million
TSM: Tumor stem medium
UCSF: University of California, San Francisco
UCSC: University of California, Santa Cruz
XMP: Xanthosine monophosphate

## References

[1] Allison AC, Almquist SJ, Muller CD, Eugui EM (1991). In vitro immunosuppressive effects of mycophenolic acid and an ester pro-drug, RS-61443. Transplant Proc.

[2] Allison AC, Eugui EM (1993). The design and development of an immunosuppressive drug, mycophenolate mofetil. Springer Semin Immunopathol.

[3] Allison AC, Eugui EM (2000). Mycophenolate mofetil and its mechanisms of action. Immunopharmacology.

[4] Allison AC, Hovi T, Watts RW, Webster AD (1975). Immunological observations on patients with Lesch-Nyhan syndrome, and on the role of de-novo purine synthesis in lymphocyte transformation. Lancet.

[5] Anastas JN, Zee BM, Kalin JH, Kim M, Guo R, Alexandrescu S (2019). Re-programing chromatin with a bifunctional LSD1/HDAC inhibitor induces therapeutic differentiation in DIPG. Cancer Cell.

[6] Aoki Y, Hashizume R, Ozawa T, Banerjee A, Prados M, James CD (2012). An experimental xenograft mouse model of diffuse pontine glioma designed for therapeutic testing. J Neurooncol.

[7] Barfeld SJ, Fazli L, Persson M, Marjavaara L, Urbanucci A, Kaukoniemi KM (2015). Myc-dependent purine biosynthesis affects nucleolar stress and therapy response in prostate cancer. Oncotarget.

[8] Barsotti C, Pesi R, Giannecchini M, Ipata PL (2005). Evidence for the involvement of cytosolic 5′-nucleotidase (cN-II) in the synthesis of guanine nucleotides from xanthosine. J Biol Chem.

[9] Barton KL, Misuraca K, Cordero F, Dobrikova E, Min HD, Gromeier M (2013). PD-0332991, a CDK4/6 inhibitor, significantly prolongs survival in a genetically engineered mouse model of brainstem glioma. PLoS One.

[10] Benjanuwattra J, Chaiyawat P, Pruksakorn D, Koonrungsesomboon N (2020). Therapeutic potential and molecular mechanisms of mycophenolic acid as an anticancer agent. Eur J Pharmacol.

[11] Carr SF, Papp E, Wu JC, Natsumeda Y (1993). Characterization of human type I and type II IMP dehydrogenases. J Biol Chem.

[12] Chen B, Ma L, Paik H, Sirota M, Wei W, Chua MS (2017). Reversal of cancer gene expression correlates with drug efficacy and reveals therapeutic targets. Nat Commun.

[13] Chen B, Wei W, Ma L, Yang B, Gill RM, Chua MS (2017). Computational discovery of niclosamide ethanolamine, a repurposed drug candidate that reduces growth of hepatocellular carcinoma cells in vitro and in mice by inhibiting cell division cycle 37 signaling. Gastroenterology.

[14] Chen EY, Tan CM, Kou Y, Duan Q, Wang Z, Meirelles GV (2013). Enrichr: interactive and collaborative HTML5 gene list enrichment analysis tool. BMC Bioinform.

[15] Chen Y, Sun L, Xu H, Dong M, Mizuno T, Vinks AA (2020). PK/PD Study of mycophenolate mofetil in children with systemic lupus erythematosus to inform model-based precision dosing. Front Pharmacol.

[16] Chong YC, Toh TB, Chan Z, Lin QXX, Thng DKH, Hooi L (2020). Targeted inhibition of purine metabolism is effective in suppressing hepatocellular carcinoma progression. Hepatol Commun.

[17] Cohen KJ, Jabado N, Grill J (2017). Diffuse intrinsic pontine gliomas-current management and new biologic insights. Is there a glimmer of hope?. Neuro Oncol.

[18] Corsello SM, Bittker JA, Liu Z, Gould J, McCarren P, Hirschman JE (2017). The drug repurposing hub: a next-generation drug library and information resource. Nat Med.

[19] DeWire M, Fuller C, Hummel TR, Chow LML, Salloum R, de Blank P (2020). A phase I/II study of ribociclib following radiation therapy in children with newly diagnosed diffuse intrinsic pontine glioma (DIPG). J Neurooncol.

[20] Dobin A, Davis CA, Schlesinger F, Drenkow J, Zaleski C, Jha S (2013). STAR: ultrafast universal RNA-seq aligner. Bioinformatics.

[21] Downing HJ, Pirmohamed M, Beresford MW, Smyth RL (2013). Paediatric use of mycophenolate mofetil. Br J Clin Pharmacol.

[22] Filbin MG, Tirosh I, Hovestadt V, Shaw ML, Escalante LE, Mathewson ND (2018). Developmental and oncogenic programs in H3K27M gliomas dissected by single-cell RNA-seq. Science.

[23] Gaffo AL, McManus LM, Mitchell RN (2014). Crystal diseases. Pathobiology of human disease.

[24] Gandolfo MT, Jang HR, Bagnasco SM, Ko GJ, Agreda P, Soloski MJ (2010). Mycophenolate mofetil modifies kidney tubular injury and Foxp3+ regulatory T cell trafficking during recovery from experimental ischemia-reperfusion. Transpl Immunol.

[25] Grasso CS, Tang Y, Truffaux N, Berlow NE, Liu L, Debily MA (2015). Functionally defined therapeutic targets in diffuse intrinsic pontine glioma. Nat Med.

[26] Hashizume R, Andor N, Ihara Y, Lerner R, Gan H, Chen X (2014). Pharmacologic inhibition of histone demethylation as a therapy for pediatric brainstem glioma. Nat Med.

[27] Hoffman LM, Veldhuijzen van Zanten SEM, Colditz N, Baugh J, Chaney B, Hoffmann M (2018). Clinical, radiologic, pathologic, and molecular characteristics of long-term survivors of diffuse intrinsic pontine glioma (DIPG): a collaborative report from the international and european society for pediatric oncology DIPG registries. J Clin Oncol.

[28] Katagi H, Louis N, Unruh D, Sasaki T, He X, Zhang A (2019). Radiosensitization by histone H3 demethylase inhibition in diffuse intrinsic pontine glioma. Clin Cancer Res.

[29] Katagi H, Takata N, Aoi Y, Zhang Y, Rendleman EJ, Blyth GT (2021). Therapeutic targeting of transcriptional elongation in diffuse intrinsic pontine glioma. Neuro Oncol.

[30] Khuong-Quang DA, Buczkowicz P, Rakopoulos P, Liu XY, Fontebasso AM, Bouffet E (2012). K27M mutation in histone H3.3 defines clinically and biologically distinct subgroups of pediatric diffuse intrinsic pontine gliomas. Acta Neuropathol.

[31] Kofuji S, Hirayama A, Eberhardt AO, Kawaguchi R, Sugiura Y, Sampetrean O (2019). IMP dehydrogenase-2 drives aberrant nucleolar activity and promotes tumorigenesis in glioblastoma. Nat Cell Biol.

[32] Li B, Dewey CN (2011). RSEM: accurate transcript quantification from RNA-Seq data with or without a reference genome. BMC Bioinform.

[33] Lin GL, Wilson KM, Ceribelli M, Stanton BZ, Woo PJ, Kreimer S (2019). Therapeutic strategies for diffuse midline glioma from high-throughput combination drug screening. Sci Transl Med.

[34] Mackay A, Burford A, Carvalho D, Izquierdo E, Fazal-Salom J, Taylor KR (2017). Integrated molecular meta-analysis of 1,000 pediatric high-grade and diffuse intrinsic pontine glioma. Cancer Cell.

[35] Misek SA, Newbury PA, Chekalin E, Paithankar S, Doseff AI, Chen B (2022). Ibrutinib blocks YAP1 activation and reverses BRAF inhibitor resistance in melanoma cells. Mol Pharmacol.

[36] Morris PG, Correa DD, Yahalom J, Raizer JJ, Schiff D, Grant B (2013). Rituximab, methotrexate, procarbazine, and vincristine followed by consolidation reduced-dose whole-brain radiotherapy and cytarabine in newly diagnosed primary CNS lymphoma: final results and long-term outcome. J Clin Oncol.

[37] Pathania M, De Jay N, Maestro N, Harutyunyan AS, Nitarska J, Pahlavan P (2017). H3.3(K27M) cooperates with Trp53 loss and PDGFRA gain in mouse embryonic neural progenitor cells to induce invasive high-grade gliomas. Cancer Cell.

[38] Paugh BS, Broniscer A, Qu C, Miller CP, Zhang J, Tatevossian RG (2011). Genome-wide analyses identify recurrent amplifications of receptor tyrosine kinases and cell-cycle regulatory genes in diffuse intrinsic pontine glioma. J Clin Oncol.

[39] Pessetto ZY, Chen B, Alturkmani H, Hyter S, Flynn CA, Baltezor M (2017). In silico and in vitro drug screening identifies new therapeutic approaches for Ewing sarcoma. Oncotarget.

[40] Piunti A, Hashizume R, Morgan MA, Bartom ET, Horbinski CM, Marshall SA (2017). Therapeutic targeting of polycomb and BET bromodomain proteins in diffuse intrinsic pontine gliomas. Nat Med.

[41] Pollack IF, Agnihotri S, Broniscer A (2019). Childhood brain tumors: current management, biological insights, and future directions. J Neurosurg Pediatr.

[42] Qin Y, Zheng X, Gao W, Wang B, Wu Y (2021). Tumor microenvironment and immune-related therapies of head and neck squamous cell carcinoma. Mol Ther Oncolytics.

[43] Ramaswamy V, Remke M, Taylor MD (2014). An epigenetic therapy for diffuse intrinsic pontine gliomas. Nat Med.

[44] Risso D, Ngai J, Speed TP, Dudoit S (2014). Normalization of RNA-seq data using factor analysis of control genes or samples. Nat Biotechnol.

[45] Robinson MD, McCarthy DJ, Smyth GK (2010). edgeR: a Bioconductor package for differential expression analysis of digital gene expression data. Bioinformatics.

[46] Rodríguez-Pascual J, Sha P, García-García E, Rajeshkumar NV, De Vicente E, Quijano Y (2013). A preclinical and clinical study of mycophenolate mofetil in pancreatic cancer. Invest New Drugs.

[47] Saratsis AM, Kambhampati M, Snyder K, Yadavilli S, Devaney JM, Harmon B (2014). Comparative multidimensional molecular analyses of pediatric diffuse intrinsic pontine glioma reveals distinct molecular subtypes. Acta Neuropathol.

[48] Sasaki T, Katagi H, Goldman S, Becher OJ, Hashizume R (2020). Convection-enhanced delivery of enhancer of zeste homolog-2 (EZH2) inhibitor for the treatment of diffuse intrinsic pontine glioma. Neurosurgery.

[49] Schmidt F, Rieger J, Wischhusen J, Naumann U, Weller M (2001). Glioma cell sensitivity to topotecan: the role of p53 and topotecan-induced DNA damage. Eur J Pharmacol.

[50] Schwartzentruber J, Korshunov A, Liu XY, Jones DT, Pfaff E, Jacob K (2012). Driver mutations in histone H3.3 and chromatin remodelling genes in paediatric glioblastoma. Nature.

[51] Serwer L, Hashizume R, Ozawa T, James CD (2010). Systemic and local drug delivery for treating diseases of the central nervous system in rodent models. J Vis Exp.

[52] Shireman JM, Atashi F, Lee G, Ali ES, Saathoff MR, Park CH (2021). De novo purine biosynthesis is a major driver of chemoresistance in glioblastoma. Brain.

[53] Stone TW, Simmonds HA (1991). Metabolism of endogenous purines. Purines: basic and clinical aspects.

[54] Subramanian A, Tamayo P, Mootha VK, Mukherjee S, Ebert BL, Gillette MA (2005). Gene set enrichment analysis: a knowledge-based approach for interpreting genome-wide expression profiles. Proc Natl Acad Sci U S A.

[55] Takebe N, Cheng X, Fandy TE, Srivastava RK, Wu S, Shankar S (2006). IMP dehydrogenase inhibitor mycophenolate mofetil induces caspase-dependent apoptosis and cell cycle inhibition in multiple myeloma cells. Mol Cancer Ther.

[56] Taubes A, Nova P, Zalocusky KA, Kosti I, Bicak M, Zilberter MY (2021). Experimental and real-world evidence supporting the computational repurposing of bumetanide for APOE4-related Alzheimer’s disease. Nat Aging.

[57] Traut TW (1994). Physiological concentrations of purines and pyrimidines. Mol Cell Biochem.

[58] Vivian J, Rao AA, Nothaft FA, Ketchum C, Armstrong J, Novak A (2017). Toil enables reproducible, open source, big biomedical data analyses. Nat Biotechnol.

[59] Wang Q, Guan YF, Hancock SE, Wahi K, van Geldermalsen M, Zhang BK (2021). Inhibition of guanosine monophosphate synthetase (GMPS) blocks glutamine metabolism and prostate cancer growth. J Pathol.

[60] Wu G, Broniscer A, McEachron TA, Lu C, Paugh BS, Becksfort J (2012). Somatic histone H3 alterations in pediatric diffuse intrinsic pontine gliomas and non-brainstem glioblastomas. Nat Genet.

[61] Wu G, Diaz AK, Paugh BS, Rankin SL, Ju B, Li Y (2014). The genomic landscape of diffuse intrinsic pontine glioma and pediatric non-brainstem high-grade glioma. Nat Genet.

[62] Wu J, Jaar BG, Briggs WA, Choi MJ, Kraus ES, Racusen LC (2004). High-dose mycophenolate mofetil in the treatment of posttransplant glomerular disease in the allograft: a case series. Nephron Clin Pract.

[63] Zeng B, Glicksberg BS, Newbury P, Chekalin E, Xing J, Liu K (2021). OCTAD: an open workspace for virtually screening therapeutics targeting precise cancer patient groups using gene expression features. Nat Protoc.

[64] Zeng WZD, Glicksberg BS, Li Y, Chen B (2019). Selecting precise reference normal tissue samples for cancer research using a deep learning approach. BMC Med Genomics.

[65] Zhou W, Yao Y, Scott AJ, Wilder-Romans K, Dresser JJ, Werner CK (2020). Purine metabolism regulates DNA repair and therapy resistance in glioblastoma. Nat Commun.

