# Supplementary material for: Reversal of cancer gene expression identifies repurposed drugs for diffuse intrinsic pontine glioma
Source: Acta Neuropathol Commun. 2022 Oct 23;10:150. doi: 10.1186/s40478-022-01463-z (PMC9590174; doi:10.1186/s40478-022-01463-z)
Supplement: Supplementary file 4 — Additional file 4: Supplementary Figures S1. Enriched target class of TOP (top) and HDAC (bottom) in the predictions. Supplementary Figures S2. Cell viability assay valuation of MMF. Supplementary Figures S3. RNAseq analysis of MMF-treated SF8628 (A) and SU-DIPG- IV (B) cells. Supplementary Figures S4. Expression of IMPDH2 in pediatric high-grade glioma cells. Supplementary Figures S5. In vivo anti-tumor activity of MMF by CED in orthotopic patient-derived DIPG xenografts. [file 40478_2022_1463_MOESM4_ESM.pdf]

Supplementary Fig.S1

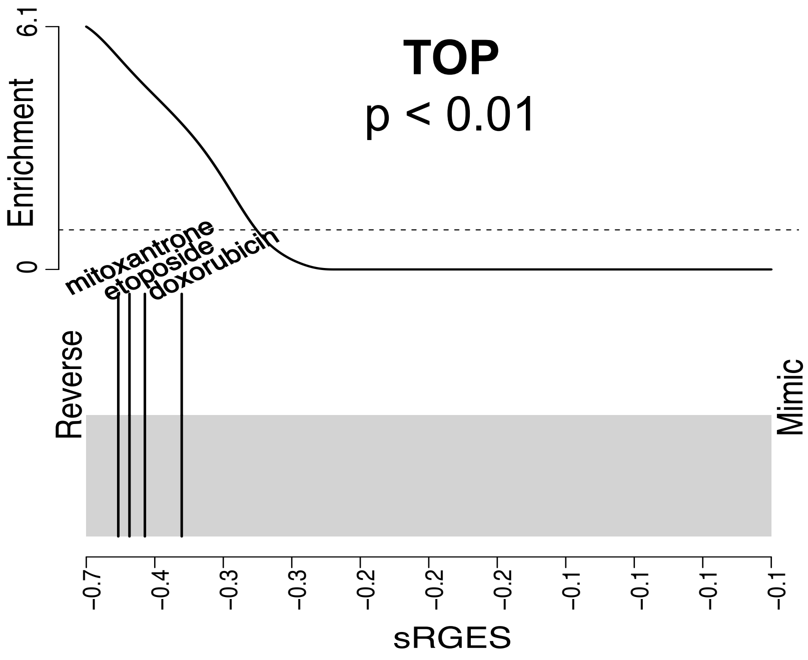

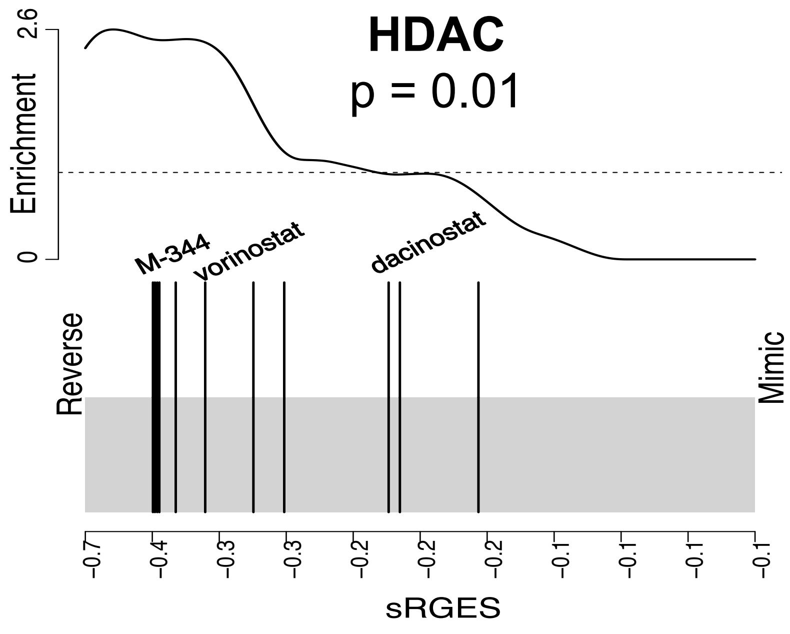

Supplementary Fig.S1 Enriched target class of TOP (top) and HDAC (bottom) in the predictions. The black line on the left suggests it is ranked on the top in the prediction list. An enriched target class means the ligands of the target tend to be highly ranked in the predictions.

Supplementary Fig.S2

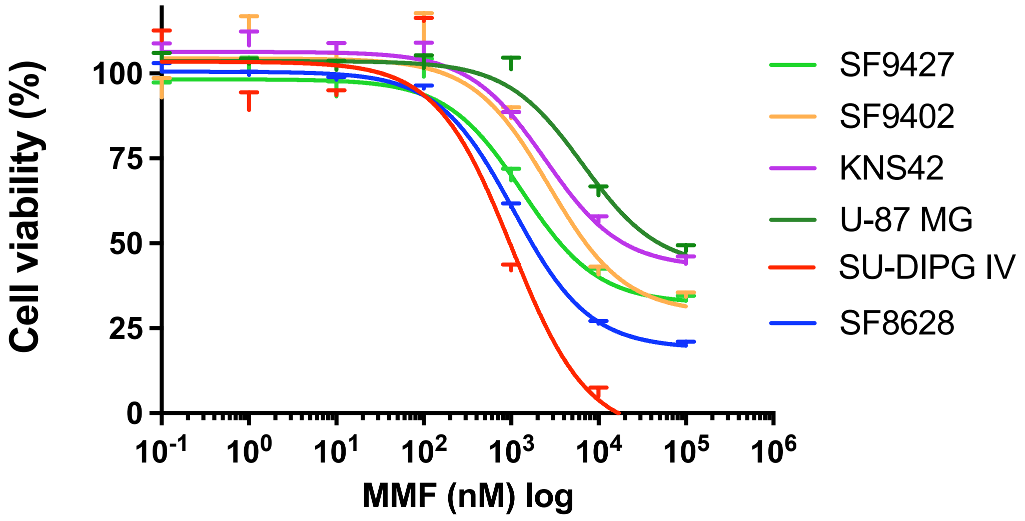

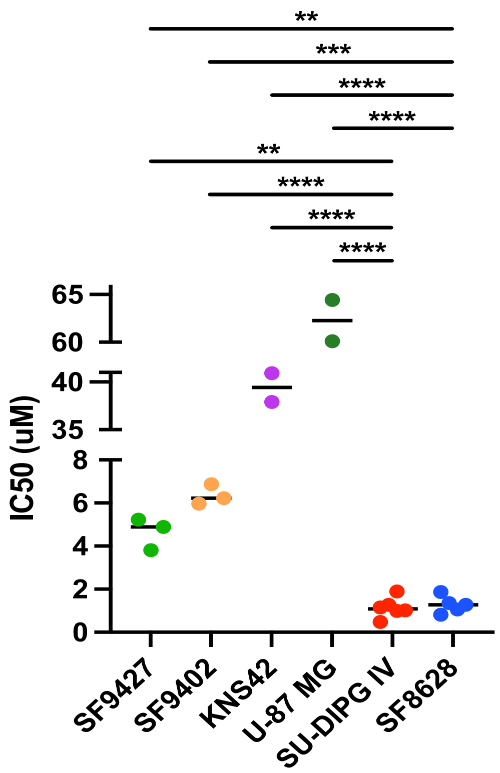

Supplementary Fig.S2 Cell viability assay valuation of MMF. MMF in H3K27M mutant DIPG cell lines (SF8628, SU-DIPG-IV), H3 wild type (SF9402, SF9427), H3G34V mutant (KNS-42), and IDH wild type (U-87 MG) GBM cell lines. Top: Graphs showing the proliferation response to increasing concentrations of each drug. Values shown are the average [mean ± standard deviation (SD)] from duplicates or triplicate samples for each incubation condition. Bottom: Dot plot representation of IC50 values for each cell lines. Statistical analysis was performed using a one-way analysis of variance: ****$P < 0.0001$; ***$P = 0.0001$; SU-DIPG IV vs. SF9427, **$P = 0.0036$; SF8628 vs. SF9427, **$P = 0.0067$.

Supplementary Fig.S3

A.

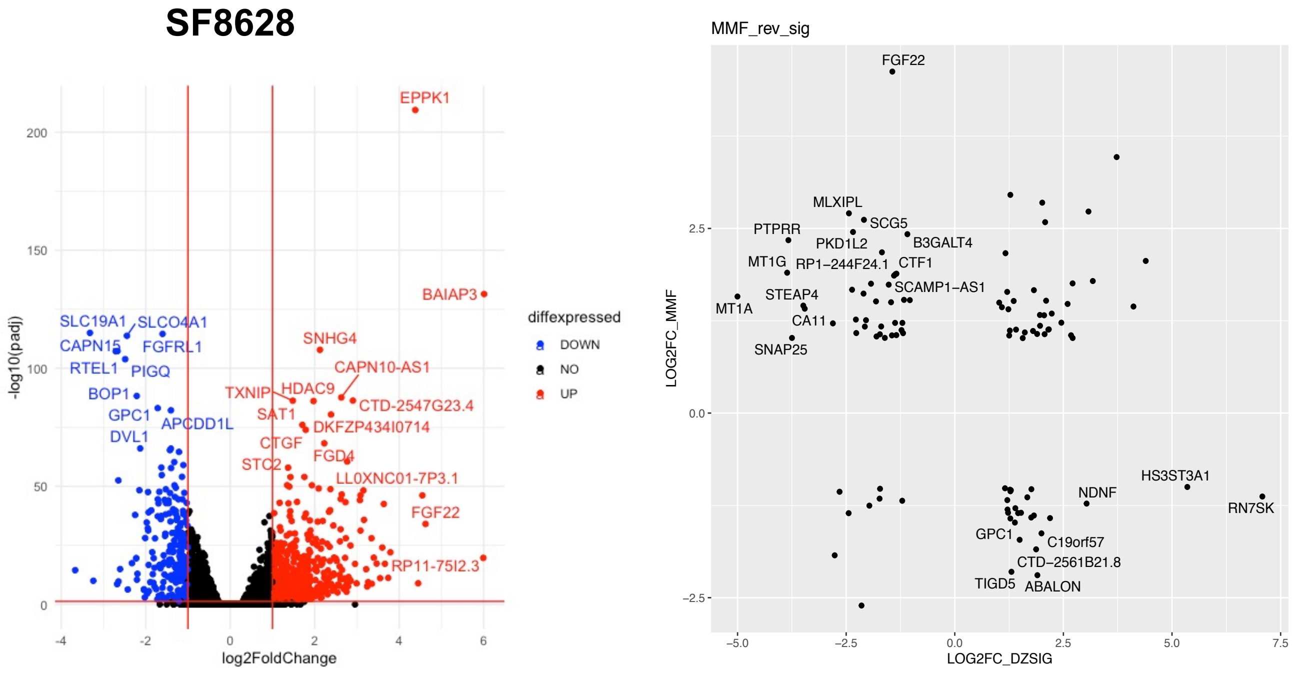

B.

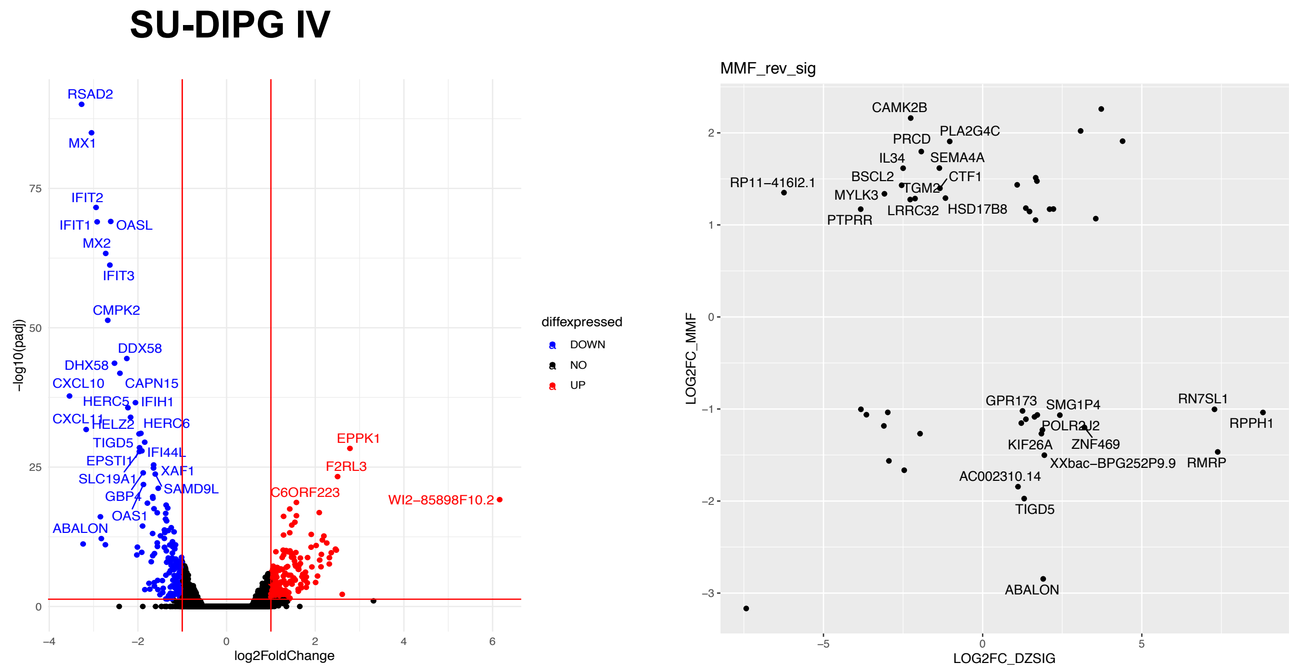

Supplementary Fig.S3 RNAseq analysis of MMF-treated SF8628 (A) and SU-DIPG-IV (B) cells. Left: Volcano plots showing differentially expressed genes. Each dot represents a gene and color indicates the expression pattern. Blue: Down-regulated genes, Black: Not differentially expressed genes and Red: Up-regulated genes **Right**: Plotted log2FC of genes from disease signature against log2FC of genes of MMF treated samples. Genes showing reversal pattern are labelled.

Supplementary Fig.S4

H3K27 status	WT	WT		H3.3K27M	H3.1K27M	H3.3K27M	WT GBM	H3.3K27M	WT GBM	H3.3K27M	H3.3K27M	H3.3G34V GBM
Cell Line	NHA	NSC		SF8628	DIPG-IV	DIPG007	SF9427	SF7761	SF9402	DIPG1114	DIPG-XIII	KNS42
Average RNA-seq reads of IMPDH2	91.04	242.83		239.36	226.91	188.61	122.39	103.94	101.33	90.36	81.49	56.30

Supplementary Fig.S4 Expression of IMPDH2 in pediatric high-grade glioma cells. Values shown are the average [mean ± standard deviation (SD)] from triplicate samples for each cell line by RNAseq analysis.

Supplementary Fig.S5

A.

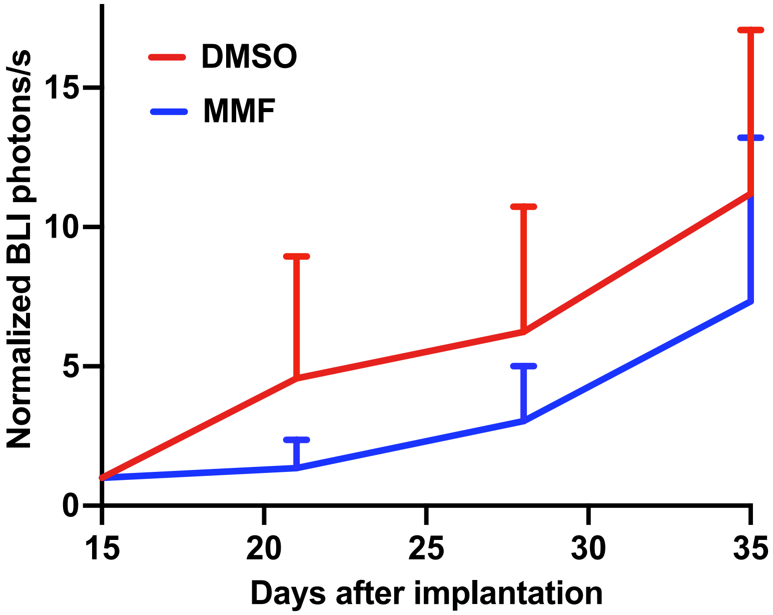

B.

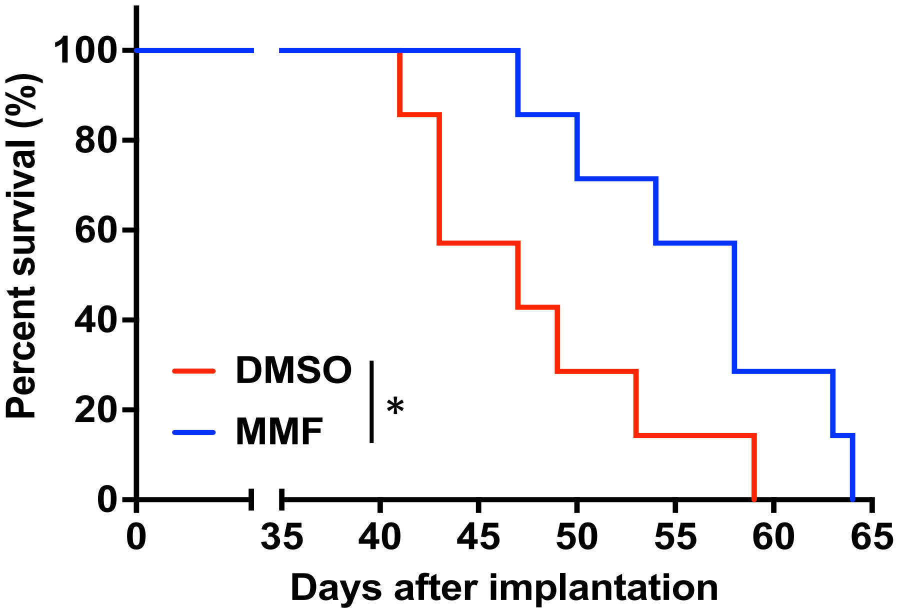

Supplementary Fig S5. *In vivo* anti-tumor activity of MMF by CED in orthotopic patient-derived DIPG xenografts. Mice bearing SF8628 intracranial (brainstem) xenografts were treated by CED with either vehicle (DMSO, n = 7) or MMF (1uM with a volume of 10uL, n = 7). (**A**) Growth curve for brainstem tumor using bioluminescence imaging in each treatment group. Tumor bioluminescence values show mean and upper SD. (**B**) Corresponding survival plots of each treatment group: **P* = 0.0439.
